# Construction of New Active Sites: Cu Substitution Enabled Surface Frustrated Lewis Pairs over Calcium Hydroxyapatite for CO_2_ Hydrogenation

**DOI:** 10.1002/advs.202101382

**Published:** 2021-07-08

**Authors:** Jiuli Guo, Yan Liang, Rui Song, Joel Y. Y. Loh, Nazir P. Kherani, Wu Wang, Christian Kübel, Ying Dai, Lu Wang, Geoffrey A. Ozin

**Affiliations:** ^1^ School of Chemistry and Chemical Engineering Anyang Normal University Anyang Henan 455000 P. R. China; ^2^ Solar Fuels Group Centre for Inorganic and Polymeric Nanomaterials Department of Chemistry University of Toronto Toronto M5S 3H6 Canada; ^3^ School of Physics State Key Laboratory of Crystal Materials Shandong University Jinan Shandong 250100 P. R. China; ^4^ Department of Electrical and Computer Engineering Department of Materials Science and Engineering University of Toronto Toronto M5S 3E4 Canada; ^5^ Karlsruhe Institute of Technology (KIT) Institute of Nanotechnology (INT) and Karlsruhe Nano Micro Facility (KNMF) Hermann‐von‐Helmholtz‐Platz 1, Building 640 Eggenstein‐Leopoldshafen 76344 Germany; ^6^ Technical University Darmstadt (TUDa) Department of Materials & Earth Sciences Alarich‐Weiss‐Straße 2 Darmstadt 64287 Germany; ^7^ School of Science and Engineering The Chinese University of Hong Kong (Shenzhen) Guangdong 518172 P. R. China

**Keywords:** copper, gas‐phase reactions, hydroxyapatite, surface chemistry, surface frustrated Lewis pair

## Abstract

Calcium hydroxyphosphate, Ca_10_(PO_4_)_6_(OH)_2_, is commonly known as hydroxyapatite (HAP). The acidic calcium and basic phosphate/hydroxide sites in HAP can be modified via isomorphous substitution of calcium and/or hydroxide ions to enable a cornucopia of catalyzed reactions. Herein, isomorphic substitution of Ca^2+^ ions by Cu^2+^ ions especially at very low levels of exchange created new analogs of molecular surface frustrated Lewis pairs (SFLPs) in Cu*_x_*Ca_10−_ *_x_*(PO_4_)_6_(OH)_2_, thereby boosting its performance metrics in heterogeneous CO_2_ photocatalytic hydrogenation. In situ Fourier transform infrared spectroscopy characterization and density functional theory calculations provided fundamental insights into the catalytically active SFLPs defined as proximal Lewis acidic Cu^2+^ and Lewis basic OH^−^. The photocatalytic pathway proceeds through a formate reaction intermediate, which is generated by the reaction of CO_2_ with heterolytically dissociated H_2_ on the SFLPs. Given the wealth of information thus uncovered, it is highly likely that this work will spur the further development of similar classes of materials, leading to the advancement and, ultimately, large‐scale application of photocatalytic CO_2_ reduction technologies.

## Graphical abstract

Complementary DRIFTS, UPS, in situ XPS, ^1^H MAS NMR, and DFT calculations provided insight into a new kind of SFLPs in low Cu^2+^ exchange Cu*_x_*Ca_10−_ *_x_*(PO_4_)_6_(OH)_2_. A formate reaction intermediate is identified, which is generated by the reaction of CO_2_ with heterolytically dissociated H_2_ on Ca─OH···Cu lattice pairs to form Ca─OH_2_^+^···CuH^−^.

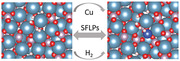

## 1 Introduction

In recent years, excessive CO_2_ emissions caused by fossil fuel combustion and automobile exhaust emissions have seriously disturbed the natural carbon cycle and exacerbated global warming. Gas‐phase photocatalytic CO_2_ reduction has attracted global attention; it aims to reduce CO_2_ into commodity chemicals and fuels exemplified by CO, CH_4_, HCOOH, CH_3_OH, and C_2+_ by using resource‐rich, economical, clean, and renewable solar energy.^[^ ^1^ ^]^ Surface frustrated Lewis pairs (SFLPs) can activate H_2_ and CO_2_ molecules due to their ability to form a highly activated structural space for adsorbed molecules. As a result, SFLPs have been implicated in gas‐phase photocatalytic CO_2_ hydrogenation to CO, CH_4_, and CH_3_OH.^[^ ^2^ ^]^

To amplify, the SFLPs sites (i.e., In─OH···In) in In_2_O_3−_ *_x_*(OH)*_y_* serve to capture and convert CO_2_ to either CO or CH_3_OH.^[^ ^2^, ^3^ ^]^ As well, oxygen vacancies were introduced in CoGeO_2_(OH)_2_ using photo‐generated holes to oxidize the hydroxyl groups of the lattice, where SFLPs involving unsaturated coordinated surface cobalt sites and adjacent hydroxyl catalyze the formation of CH_4_ from CO_2_ and H_2_O.^[^ ^4^ ^]^ Additionally, through isomorphous substitution of In^3+^ in In_2_O_3−_ *_x_*(OH)*_y_* or In_2_O_3_ with Bi^3+^, the reactivity of the SFLPs themselves in Bi_z_In_2−_ *_z_*O_3−_ *_x_*(OH)*_y_* or Bi*_x_*In_2−_ *_x_*O_3_ can be tailored to advantage in heterogeneous CO_2_ photocatalytic hydrogenation.^[^ ^5^ ^]^

Recently, we have discovered Cu^2+^ ions substitute for Ca^2+^ into the HAP lattice and hydrated Cu^2+^ species therein migrate and deposit to form CuO at the surface for high levels of exchange Cu‐HAP.^[^ ^6^ ^]^ Notably, Cu^2+^/PO_4_^3−^ SFLPs at high levels of exchange Cu‐HAP were found to greatly enhance CO_2_ reduction to CO driven photothermally.^[^ ^6^ ^]^

To understand if the Cu^2+^ in HAP structure has any relevance in the reaction, it became apparent that low levels of exchange (0 and 0.5 mol%) Cu‐HAP might be advantageous to avoid CuO in flow CO_2_ conversion to products. Amazingly, these results revealed a CO production rate of 215 µmol g_cat_^−1^ h^−1^ with no CH_4_ being detected for 0.5 mol% Cu‐HAP, which is around 61 orders photoactivity enhancement of the reverse water gas shift (RWGS) reaction compared to the pristine 0 mol% Cu‐HAP. What surprised us, even more, was the new Cu^2+^/OH^−^ SFLPs in 0.5 mol% Cu‐HAP, composed of coordinately unsaturated copper sites, adjacent to an oxygen vacancy and a hydroxide group, enable the heterolysis of H_2_ and reaction with CO_2_ to form CO through a formate reaction intermediate driven photocatalytically.

## 2 Results and Discussion

The synthesis of 0 and 0.5 mol% Cu‐HAP is accomplished by the co‐precipitation method. 0.5 mol% Cu‐HAP displayed the very similar powder X‐ray diffraction (PXRD) pattern of hexagonal (space group P6_3_/m) HAP but a small shift to lower 2*θ* diffraction angles relative to those of 0 mol% Cu‐HAP (Figure S1, Supporting Information), which signals the substitution of the larger ionic radius Ca^2+^ (1.0 Å) by smaller Cu^2+^ (0.73 Å).^[^ ^5^ ^]^ This conclusion is further supported by representative scanning transmission electron microscopy imaging in combination with energy‐dispersive X‐ray spectroscopy images of 0.5 mol% Cu‐HAP nanocrystals, where no metallic Cu or CuO*_x_* nanoparticles were observed despite a significant Cu loading (Figure S2, Supporting Information). Furthermore, electron paramagnetic resonance (EPR) signals from 0.5 mol% Cu‐HAP were recorded and confirm the Cu^2+^ sites in the HAP lattice (Figure S3, Supporting Information).^[^ ^7^ ^]^

**Figure 1:**
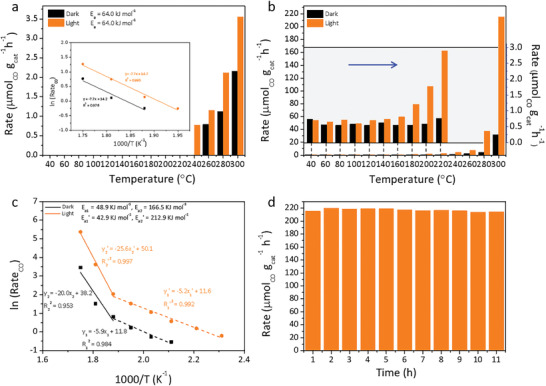
Photocatalytic performance of 0 and 0.5 mol% Cu‐HAP in the flow reactor. CO rate of a) 0 and b) 0.5 mol% Cu‐HAP in the flow reactor under dark and light conditions. Arrhenius plots for CO production rates of 0 mol% Cu‐HAP are shown inset of Figure 1a. c) Arrhenius plots for CO production rates of 0.5 mol% Cu‐HAP. d) Catalytic stability results for 0.5 mol% Cu‐HAP in a flow reactor over 11 h at 300 °C under light conditions. Reaction conditions for flow measurements: atmospheric pressure, light intensity of ≈2.0 W cm^−2^, H_2_/CO_2_ ratio = 1:1 with flow rate of 2 sccm.

Samples were tested at different temperatures in the flow reactor with and without light irradiation. As shown in **Figure** **1**a,b, 0.5 mol% Cu‐HAP was more active than 0 mol% Cu‐HAP to catalyze RWGS reaction, CO_2_ + H_2_ → CO + H_2_O towards the sole production CO detected (confirmed by ^13^CO_2_ isotope labeling in Figure S4, Supporting Information). Notably, 0.5 mol% Cu‐HAP showed higher CO rate of 215 μmol_CO_ g_cat_^−1^ h^−1^ at 300 °C under light than 0 mol% Cu‐HAP by around 61 orders of magnitude. The linear Arrhenius behavior for 0 mol% Cu‐HAP suggests that a single catalytic process occurs within the temperature range of the study under both dark and light (inset of Figure 1a). Surprisingly, the corresponding Arrhenius plots for 0.5 mol% Cu‐HAP showed two‐stage linear plots with activation energies of 48.9 kJ mol^−1^ (low temperature, 200–260 °C) and 166.5 kJ mol^−1^ (high temperature, 260–300 °C) under dark, and 42.9 kJ mol^−1^ (low temperature, 160–260 °C) and 212.9 kJ mol^−1^ (high temperature, 260–300 °C) with light (Figure 1c), indicating two different reaction pathways.^[^ ^8^ ^]^ The control test revealed no activity in blank reactor with quartz wool at 300 °C and light illumination.

An 11‐h stability of 0.5 mol% Cu‐HAP was investigated in the capillary flow reactor at 300 °C with light (Figure 1d), and showed a decrease in production rate of only 0.5%. It is noteworthy that the PXRD pattern of the spent 0.5 mol% Cu‐HAP indicated the intact hydroxyapatite (HAP) lattice and no copper nanocrystal was observed (Figure S5), which was further supported by that no visible change for its light blue color.

Operando Diffuse Reflectance Infrared Fourier Transform Spectroscopy (DRIFTS) is a powerful technique for observing surface species participating in catalytic reactions under real‐world conditions. The DRIFTS method was therefore applied to study the RWGS reaction photocatalyzed by 0 and 0.5 mol% Cu‐HAP, the goal being to identify fingerprint vibrational modes diagnostic of adsorbed reactants and intermediates that exist as a function of temperature and time.

**Figure 2:**
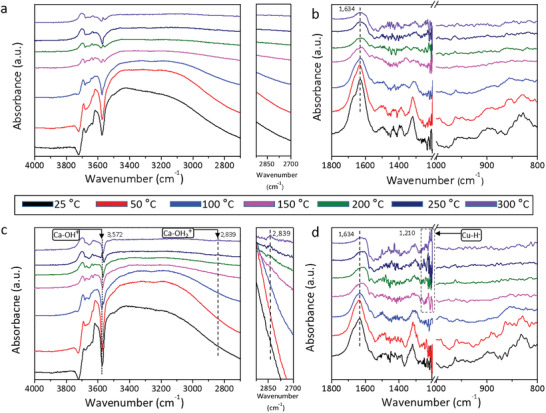
DRIFTS results for 0 and 0.5 mol% Cu‐HAP. DRIFTS spectra obtained during H_2_ (1 sccm H_2_, 19 sccm He) adsorption on a,b) 0 and c,d) 0.5 mol% Cu‐HAP after 30 min at each temperature.

Operando DRIFTS experiments were performed to gain an insight into the RWGS reaction catalyzed by 0 and 0.5 mol% Cu‐HAP. One type involves exposing the catalysts to H_2_ (**Figure** **2**), these experiments were conducted as a function of temperature and time to provide information on adsorbed surface hydrogen species. The surprising observation revealed that the H_2_ molecule reacts with 0.5 mol% Cu‐HAP to produce new vibrational modes in the hydroxyl and metal hydride stretching and deformation regions with increased temperature, in which the 0 mol% Cu‐HAP showed nothing. In detail, the peaks at 1210 and 2839 cm^−1^ can be assigned to Cu‐H^−^ and Ca‐OH_2_^+^, respectively.^[^ ^2^, ^9^ ^]^ For peak located at 3,572 cm^−1^, could be attributed to the protonated calcium oxide species (Ca‐OH^+^),^[^ ^2c^ ^]^ and further evidence stems from their appearance of 250 °C and disappearance of 300 °C (Figure S6, Supporting Information). Furthermore, the decreased peak intensity under He confirmed the presence of Cu‐H^−^ and Ca‐OH_2_^+^ (Figure S7, Supporting Information). The peak at 1,634 cm^−1^ can be ascribed to adsorbed water.^[^ ^9b^ ^]^ No HPO_4_^2−^ peaks were observed at 3235, 1235, 831 cm^−1^ at high temperatures, indicating PO_4_^3−^ was not involved in the hydrogen activation on SFLPs.^[^ ^6^, ^10^ ^]^ These results indicate that H_2_ is undergoing heterolysis on proximal Lewis acidic Cu^2+^ and Lewis basic OH^−^ SFLPs to form CuH^−^/Ca‐OH_2_^+^ surface sites.

**Figure 3:**
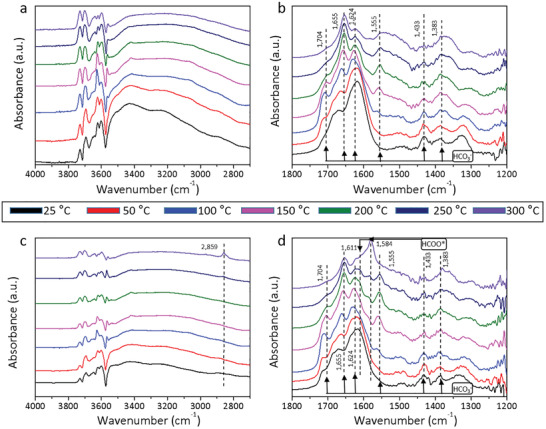
DRIFTS studies of 0 and 0.5 mol% Cu‐HAP. H_2_ and CO_2_ (1 sccm H_2_, 1 sccm CO_2_, 18 sccm He) adsorption spectra of a,b) 0 and c,d) 0.5 mol% Cu‐HAP after 30 min at different temperatures.

Further operando DRIFT study was conducted and explored the reaction of catalysts with H_2_‐CO_2_ mixtures under flow conditions after aforementioned H_2_ absorption, probed as a function of temperature and time (**Figure** **3**). Significantly, CuH^−^ and Ca‐OH_2_^+^ peaks were replaced by those of reaction intermediates gradually changed from bicarbonate to formate with increasing temperature, consistent with the observations of two‐stage linear plots in Arrhenius plots of 0.5 mol% Cu‐HAP (Figure 1c). The peaks located at 1704, 1655, 1624, 1555, 1433, and 1383 cm^−1^ can be attributed to bicarbonate intermediates.^[^ ^6^, ^9^, ^11^ ^]^ For peaks at 2859, 1611, and 1584 cm^−1^, correspond to vibrational modes of formate species.^[^ ^6^, ^11^, ^12^ ^]^ The decrease of CO_2_ peak (3600–3800 cm^−1^) intensity reflected an enhanced CO_2_ activation over 0.5 mol% Cu‐HAP with Cu^2+^ substitution.^[^ ^2^, ^6^ ^]^ The growth of intense vibrational modes in hydroxyl stretching and deformation region also confirmed the enhanced CO_2_ activation, as well as the higher H_2_ adsorption capacity (Figure S8, Supporting Information).

X‐ray photoelectron spectroscopy (XPS) spectra were acquired during exposure of 0.5 mol% Cu‐HAP to 1:1 ratio of CO_2_/H_2_ atmosphere at 300 °C under light (Figure S9, Supporting Information). A positive shift for O 1s ionization potentials of the hydroxyl flags the H_2_ heterolysis on the surface of 0.5 mol% Cu‐HAP, along with the generation of additional hydroxides and protonation.^[^ ^3b^ ^]^ Concurrently, the Cu 2p XPS peak slightly shifted to lower energy due to the formation of hydrides by H_2_ heterolysis, resulting in the low effective nuclear charge at coordinately unsaturated copper Lewis acid sites.^[^ ^3b^ ^]^ Furthermore, surface Cu atomic percentages in 0.5 mol% Cu‐HAP before and after photocatalytic reaction (1 h) did not show a significant change, which were determined to be 0.07% and 0.06% via XPS analysis, respectively (Figure S9b, Supporting Information). Solid‐state ^1^H MAS‐NMR spectra of 0.5 mol% Cu‐HAP treated before and after exposing to H_2_ at atmospheric pressure and 300 °C provide additional support for H_2_ heterolysis on SFLPs Ca─OH···Cu sites (Figure S10, Supporting Information). Two new chemical shifts around 8.23 and 12.33 ppm can be assigned as Cu‐H^−^ and Ca‐OH_2_^+^ sites, respectively.^[^ ^3^, ^13^ ^]^

**Figure 4:**
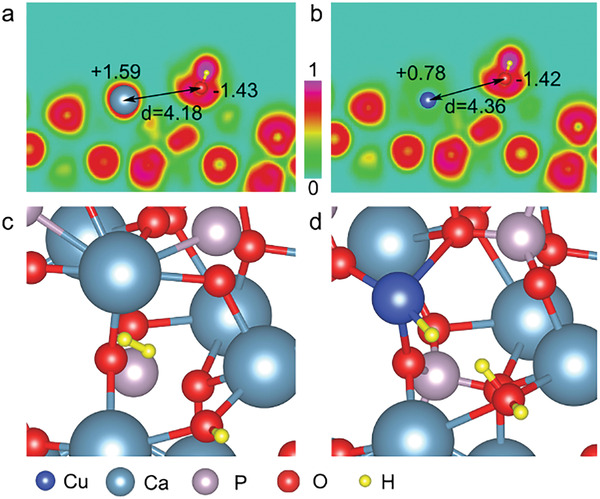
SFLPs on a) 0 mol% Cu‐HAP (211) and b) 0.5 mol% Cu‐HAP (211), where the background denotes the electron localization. Configurations of H_2_ adsorption at the c) Ca─OH···Ca SFLPs site in 0 mol% Cu‐HAP (211) and d) Ca─OH···Cu SFLPs site in 0.5 mol% Cu‐HAP (211).

DFT calculations were carried out to further reveal the promotion effect of Cu^2+^ substitution on HAP. The HAP (211) facet has been chosen for the calculation and explanation for its low average surface energy (Table S1, Supporting Information). Then, we studied the possible SFLPs site in HAP (211) with/without Cu substitution. Intrinsic OH group and metal atom at the (211) surface of HAP act as Lewis basic and Lewis acidic sites, respectively. After surface relaxation and leave out pairs where the distances are too close or too far, we screen out one possible SFLPs in pure and single‐site Cu substituted HAP (211). O and Ca pair include atomic local charges of +1.59 e and −1.43 e in pure HAP (211), and +0.78 e, −1.42 e for O atom and the near Cu atom are involved in single‐site Cu substituted HAP (211). The distances between the active O atom and Ca/Cu atom are 4.18 and 4.36 Å, respectively (**Figure** **4**a,b). These results indicated surface OH group and Ca/Cu are suitable for SFLPs.^[^ ^5b^ ^]^ On the other hand, to study the origin of the observed improvement of CO_2_ hydrogenation, the SFLPs‐mediated rate‐determine step (H_2_ dissociation) is considered (Figure 4c,d). It is found that Cu substitution induced SFLPs to dissociate H_2_ much easier, while in pristine HAP (211), H_2_ tends to maintain as a single molecule (0 K). This behavior could result from the atomic electronic configuration where the charge localization near Cu is less favorable relative to that around the Ca (see Figure 4a,b). This can be the main factor that in turn influences the bonding between H atom and intermediate products.^[^ ^5^, ^12^ ^]^

Another point worth mentioning is that the valence band positions of 0 and 0.5 mol% Cu‐HAP were determined to be −15.84 eV and −15.33 eV (vs vacuum) by ultraviolet photoelectron spectroscopy (UPS), and conduction band positions were located at −13.08 and −12.55 eV (vs vacuum), respectively (Figures S11 and S12). Notably, photoluminescence results for 0 and 0.5 mol% Cu‐HAP show an efficient charge transfer at the interface with Cu substitution (Figure S13, Supporting Information). One can surmise that photogenerated electrons and holes would relax to mid‐gap Lewis‐acidic Cu^2+^ and Lewis‐basic OH^−^ sites of SFLPs, enhancing its Lewis acidity and Lewis basicity and thereby contribute to significantly increased catalytic rates of 0.5 mol% Cu‐HAP.^[^ ^1^, ^2^, ^5^, ^11^, ^12^ ^]^

Collecting together all of the results of this study, intended to define how Cu^2+^ in HAP structure relate to the gas‐phase heterogeneous (photo)catalytic hydrogenation of CO_2_, for low levels of replacement *x* of Ca^2+^ by Cu^2+^, one can draw the reaction pathway for the production of CO by heterogeneous hydrogenation of gaseous CO_2_ on 0.5 mol% Cu‐HAP. At low temperatures (25–≈250 °C), surface oxygen vacancies could act as active sites leading to bicarbonate intermediates for CO_2_ hydrogenation to produce CO and H_2_O.^[^ ^2^, ^14^ ^]^ While at high temperatures (≈250–300 °C), H_2_ heterolytically dissociated on SFLPs Ca─OH···Cu to form Ca─OH_2_^+^···CuH^−^, subsequent reaction of the formate intermediates with protons of Ca─OH_2_^+^ form CO and H_2_O, thereby completing the RWGS catalytic cycle (Figure S14, Supporting Information). This is consistent with the higher production of CO even at dark conditions for 0.5 mol% Cu‐HAP (31.5 μmol g_cat_^−1^ h^−1^) than 0 mol% Cu‐HAP (2.2 μmol g_cat_^−1^ h^−1^).

## 3 Conclusion

In conclusion, complementary DRIFTS, UPS, in situ XPS, ^1^H MAS NMR, and DFT calculations provided insight into a new kind of SFLPs in low Cu^2+^ exchange Cu*_x_*Ca_10−_ *_x_*(PO_4_)_6_(OH)_2_, the identities of surface intermediates and details of photocatalytic reaction pathways. A formate reaction intermediate is identified, which is generated by the reaction of CO_2_ with heterolytically dissociated H_2_ on Ca─OH···Cu lattice pairs to form Ca─OH_2_^+^···CuH^−^. This represents a new class of SFLP for gas‐phase heterogeneous (photo)catalytic hydrogenation of CO_2_. Opportunities for additional modifications to the Lewis acidic and basic properties of the HAP are possible using different cations, which bodes well for tuning the activity and selectivity of the catalyst to produce a variety of products from CO_2_.

## Conflict of Interest

The authors declare no conflict of interest.

## Supporting information

Supporting Information

## Data Availability

The data that support the findings of this study are available from the corresponding author upon reasonable request.
