# Supplementary material for: Construction of New Active Sites: Cu Substitution Enabled Surface Frustrated Lewis Pairs over Calcium Hydroxyapatite for CO2 Hydrogenation
Source: Adv Sci (Weinh). 2021 Jul 8;8(17):2101382. doi: 10.1002/advs.202101382 (PMC8425883; doi:10.1002/advs.202101382)
Supplement: Supplementary file 1 — Supporting Information [file ADVS-8-2101382-s001.pdf]

WILEY-VCH

© 2021 Wiley-VCH GmbH

ADVANCED SCIENCE Open Access

Supporting Information

for *Adv. Sci.*, DOI: 10.1002/advs.202101382

Construction of New Active Sites: Cu Substitution Enabled Surface Frustrated Lewis Pairs over Calcium Hydroxyapatite for CO_2 Hydrogenation

*Jiuli Guo,*Yan Liang, Rui Song, Joel Y. Y. Loh, Nazir P. Kherani, Wu Wang, Christian Kübel, Ying Dai,* Lu Wang,* and Geoffrey A. Ozin**

Supporting Information

Construction of New Active Sites: Cu Substitution Enabled Surface Frustrated Lewis Pairs over Calcium Hydroxyapatite for CO_2 Hydrogenation

*Jiuli Guo,*Yan Liang, Rui Song, Joel Y. Y. Loh, Nazir P. Kherani, Wu Wang, Christian Kübel, Ying Dai,* Lu Wang,* and Geoffrey A. Ozin**

Calculation of Production Rates

$$\text{CO rate } (\mu\text{mol } {g_{\text{cat}}}^{-1}\ \text{h}^{-1}) = \frac{\text{CO concentration (ppm)} \times \text{Total moles (mol)}}{\text{Catalyst mass (g)} \times \text{Total reaction time (h)}} \tag{1}$$

$$\text{CO concentration (ppm)} = \frac{\text{CO FID area}}{\text{S} \times \text{GC sample pressure (Pa)}} \tag{2}$$

$$\text{S} = \frac{\text{CO FID area}}{\text{Calibrated CO concentration (ppm)} \times \text{GC pressure (Pa)}} \tag{3}$$

$$\text{Total moles (mol)} = \frac{\text{Atmospheric pressure (Pa)} \times \text{Total volume } (\text{m}^3)}{\text{R } \times \text{Reactor temperature (K)}} \tag{4}$$

where S is a scale factor, which is obtained through GC calibration using standardized gas concentration; R = 8.314 J mol^{-1} K^{-1} is the ideal gas constant.

Experimental Section

Synthesis of Cu-HAP samples: 0.5 mol% Cu-HAP were synthesized via a co-precipitation method reported before.[1] 1.175 g of $Ca(NO_3)_2\cdot4H_2O$ was dissolved in the mixed solution of 0.01 M $Cu(NO_3)_2\cdot3H_2O$ (2.5 mL) and deionized water (100 mL) and adjusted to a pH of 10-11 using 0.025 M ammonium. $(NH_4)_2HPO_4$ solution prepared by dissolving 0.396 g of $(NH_4)_2HPO_4$ in 100 mL of deionized water was then added dropwise into the previous calcium/copper solution. The resulting reaction mixture was kept in a pre-heated oil bath at 85 °C for 2 h and then aged at room temperature for 12 h before the precipitate was collected via filtration and centrifugation and washed three times with ethanol. The final product was dried overnight at 60 °C in a vacuum oven and calcined for 5 h at 500 °C. 0 mol% Cu-HAP was prepared using the same procedure without $Cu(NO_3)_2\cdot3H_2O$.

Characterization: Powder X-ray diffraction (PXRD) was performed on a Bruker D2-Phaser X-ray diffractometer using Cu Kα radiation at 30 kV. Transmission electron microscopy (TEM) analysis was performed using a FEI Titan 80-300 operated at an acceleration voltage of 300 kV, equipped with a Gatan US1000 slow-scan CCD camera, a Fischione 3000 high-angle annular dark-field (HAADF) detector, and an EDAX s-UTW energy dispersive X-ray (EDX) detector. Cu-HAP samples were prepared by dispersing the dry powder on a carbon coated Ni grid for TEM analysis. The electron paramagnetic resonance (EPR) spectra were obtained using a Bruker X-band CW EMX EPR spectrometer with a 6″ electromagnet, and an ER 4119HS resonator was applied to detect. X-ray photoelectron spectroscopy (XPS) spectra were obtained on the Thermo Scientific ESCALAB XI+ XPS Microprobe with a pressure of 4×10^{-7} Torr in an ultrahigh vacuum chamber. Photoelectron detection with a high pass energy (100 eV) and low point density (1 point eV^{-1}), and collected XPS results were calibrated to C 1s 284.5 eV. Samples for XPS were treated by H_2 and then transferred to an Ar glovebox for sample loading. Ultraviolet photoelectron spectroscopy (UPS) was performed using Thermo Escalab 250Xi instrument using monochrome Al Ka (hv =1486.6 eV), power 150W and binding energy C1s 284.8 calibration. Silver is used as reference material to calibrate Fermi edge and He I 21.22eV for UPS characterization. The 1H solid state MAS NMR measurements were performed using a JNM-ECZ400/600R with a spinning rate of 15 kHz. Samples for 1H solid state MAS NMR were treated by H_2 and then transferred to an Ar glovebox for sample loading. The photoluminescence (PL) measurements were performed at an excitation of wavelength of 340 nm using a PTI QM-4 fluorescence spectrophotometer.

In situ DRIFTS measurements: *In situ* diffuse reflectance infrared Fourier transform spectroscopy (DRIFTS) spectra were performed on a Thermo Scientific IS50 Series FT-IR instrument equipped with a Harrick Praying Mantis DRIFTS accessory and a Harrick hightemperature reaction chamber (HTC) with ZnSe windows. samples were first pretreated with 20 sccm He at 300 °C for 2 h. Background spectra with a resolution of 4 cm^{-1} and 64 scans were acquired at the relevant temperature in a 20 sccm

flow of He. Sample spectra were collected with a resolution of 4 cm^{-1} and 16 scans. A flow rate of 1 sccm H_2, 19 sccm He was used for H_2 adsorption, followed by H_2/CO_2 co-adsorption conduction in flow rates of 1 sccm H_2, 1 sccm CO_2, and 18 sccm He.

DFT calculations: The first-principles simulations were conducted using density functional theory within the generalized gradient approximation (PBE) as implemented in the vienna ab initio simulation package. The plane-wave kinetic energy threshold (400 eV), K-points were distributed using a Monkhorst-Pack-grid with a spacing of 0.03 Å^{-1}. The convergence tolerances of energy, and force were, respectively, set to be 1×10^{-5} eV per atom, 2×10^{-2} eV Å^{-1}. Starting from the relaxed bulk, 2D surface models were constructed using a periodic arrangement of slabs, separated with a 2 nm vacuum region.

Gas-phase catalytic flow reactor measurements: The photocatalytic CO_2 hydrogenation was performed in a fixed-bed tubular reactor using a borosilicate tube (with an outer diameter of 3 nm and inner diameter of 2.5 nm). 10 mg samples were packed into the borosilicate tube between two portions of quartz wool. A 300 W Newport Xe lamp with a measured intensity of ~2 W cm^{-2} was used to illuminate the catalyst and external heating was provided by conduction *via* a heated copper block. The sample was maintained at each specific temperature for 3 runs, and the total time taken to complete these 3 runs is 2 hours before advancing to the next set point at an ascent rate of 5 °C min^{-1}. During the reaction, 1 sccm H_2 (Grade 5.0) and 1 sccm CO_2 (Grade 5.0) were flowed through the reactor controlled by Alicat Scientific digital flow controllers. A SPI 8610 gas thromatograph with an FID was used to analysis product gases using He as the carrier gas.

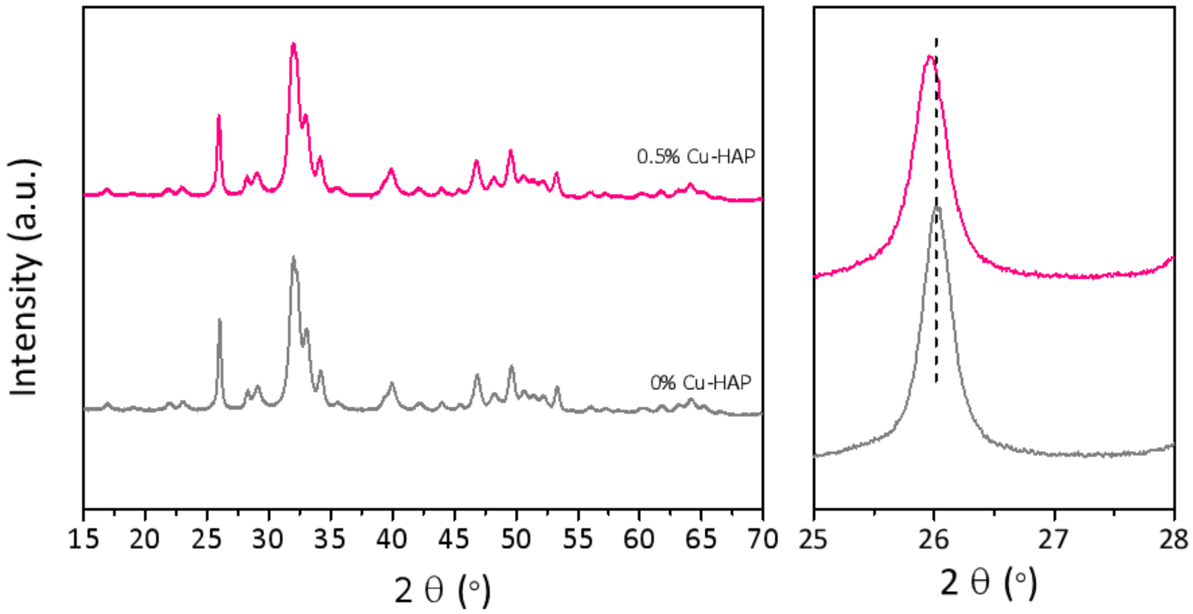

Figure S1. PXRD patterns of 0 and 0.5 mol% Cu-HAP. No metallic Cu or CuO_x peaks were observed in the PXRD pattern of 0.5 mol% Cu-HAP.

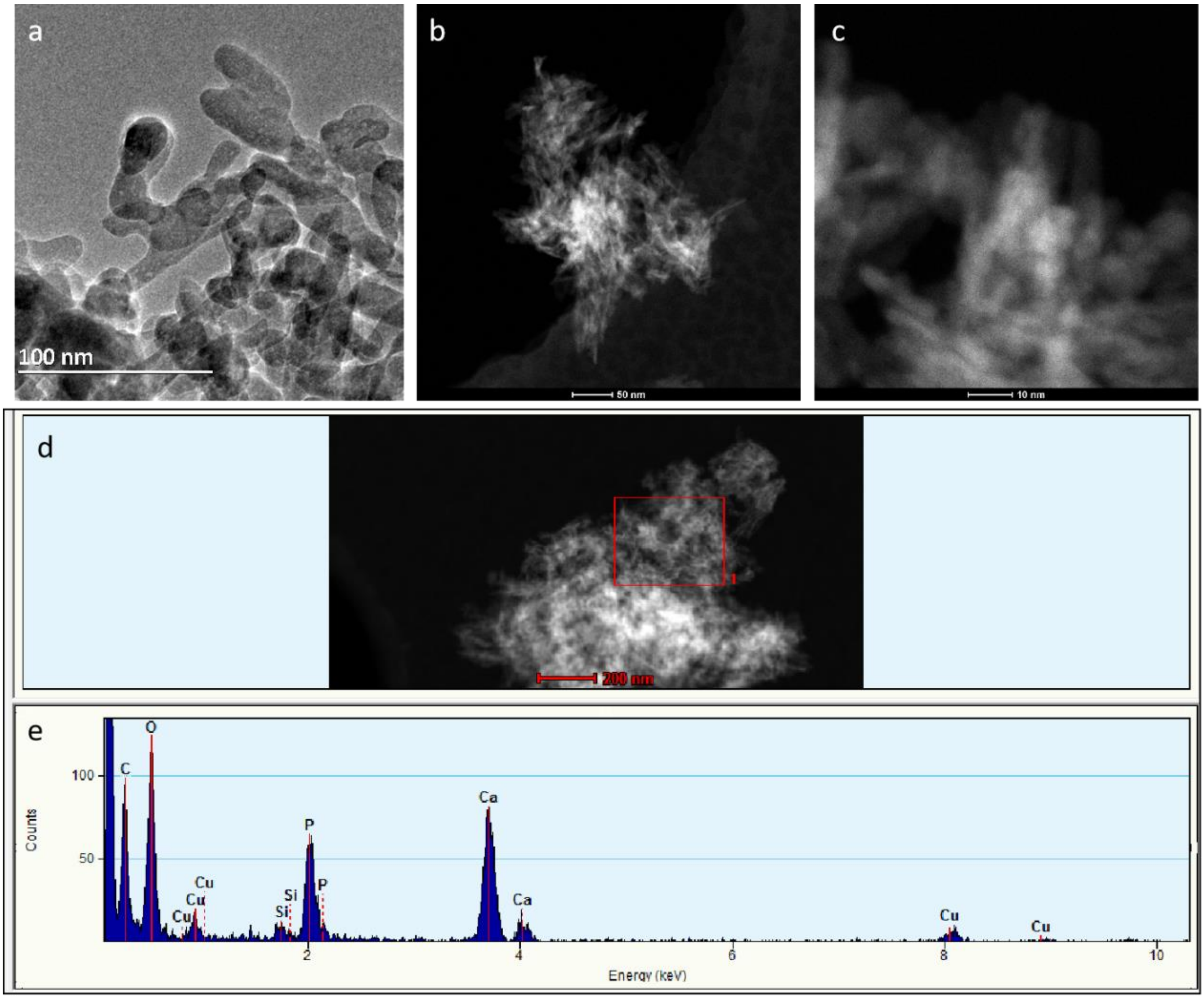

Figure S2. a) TEM image of 0 mol% Cu-HAP. b-d) HAADF-STEM images of 0.5 mol% Cu-HAP illustrating that no small Cu or CuO_x NPs are observed. e) EDX spectrum of 0.5 mol% Cu-HAP, acquired in the area indicated in d, confirming the presence of Cu, further supporting the isomorphic substitution of Ca^{2+} ions by Cu^{2+} ions.

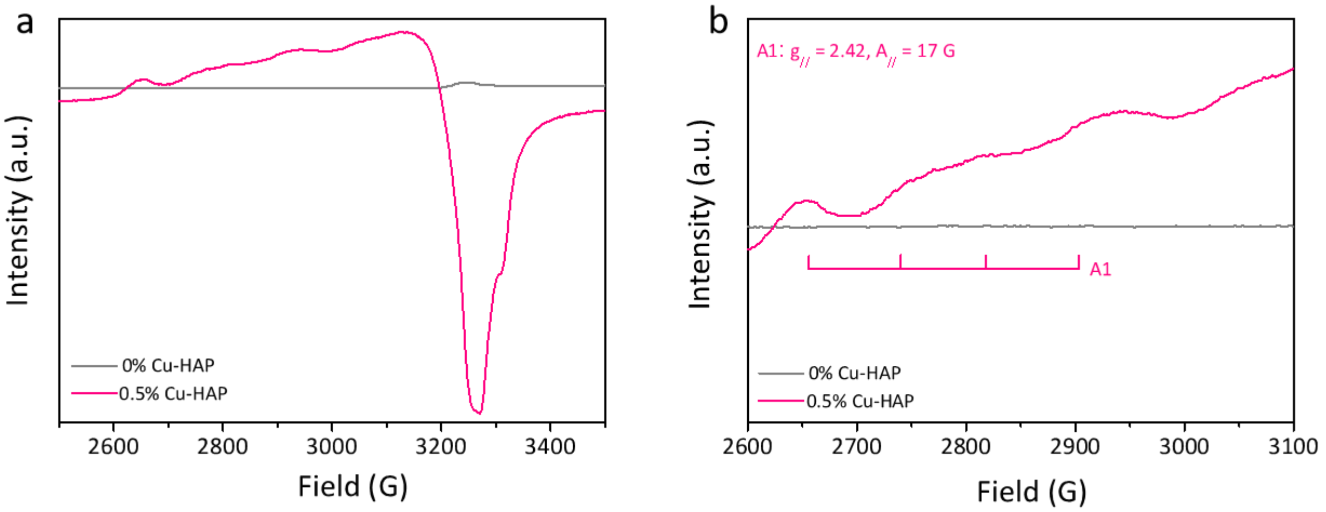

Figure S3. a) EPR spectra and b) magnified EPR spectra over the range of 2600-3100 G of 0 and 0.5 mol% Cu-HAP.

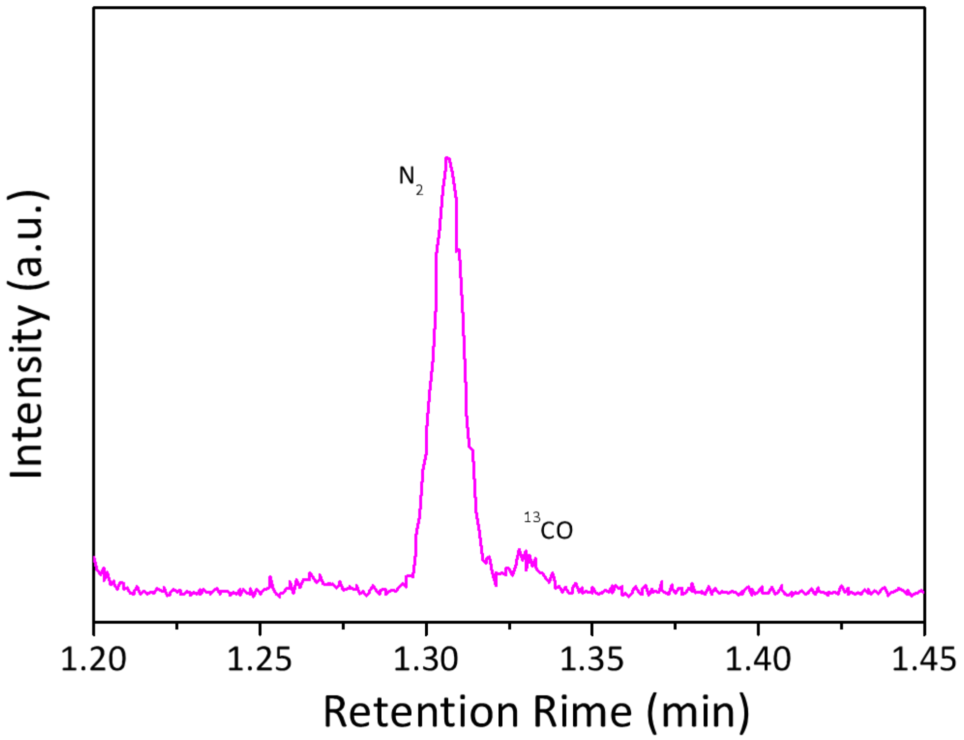

Figure S4. Mass spectrograph of 0.5 mol% Cu-HAP sample generating ^{13}CO from $^{13}CO_2$ in the batch reactor. The AMU 29 mass fragment peak at approximately 1.35 min corresponds to ^{13}CO, and peak at approximately 1.32 min corresponds to adventitious N_2. Reaction conditions for batch measurement: light intensity of 40 sun, H_2/CO_2 ratio = 1:1, measurement time of 1 h and no external heating.

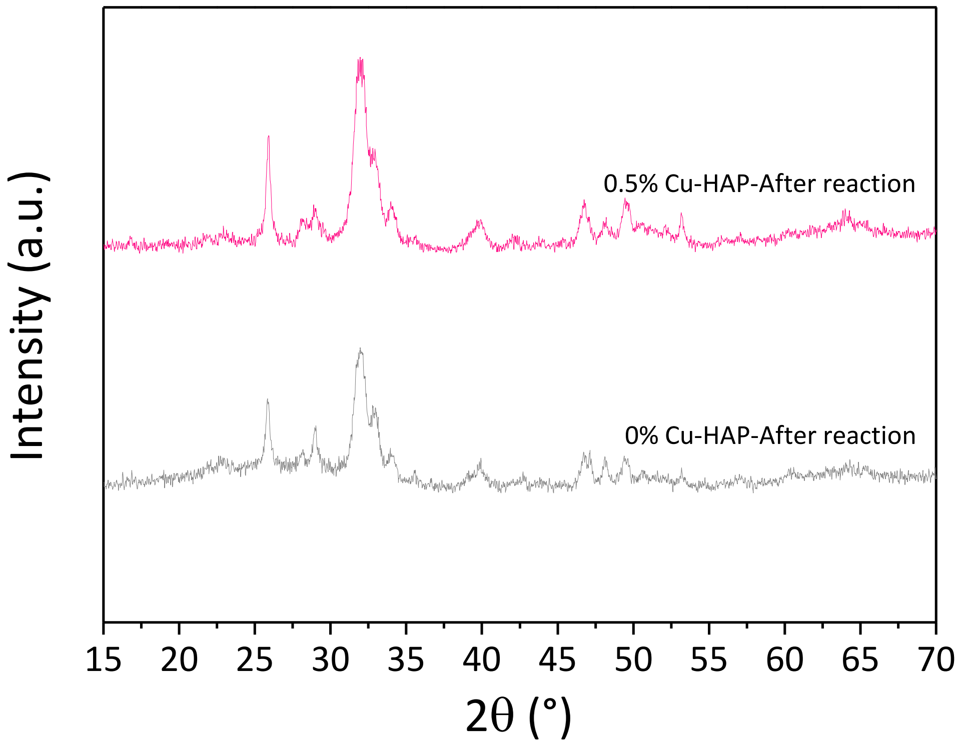

Figure S5. PXRD patterns of spent 0 and 0.5 mol% Cu-HAP samples.

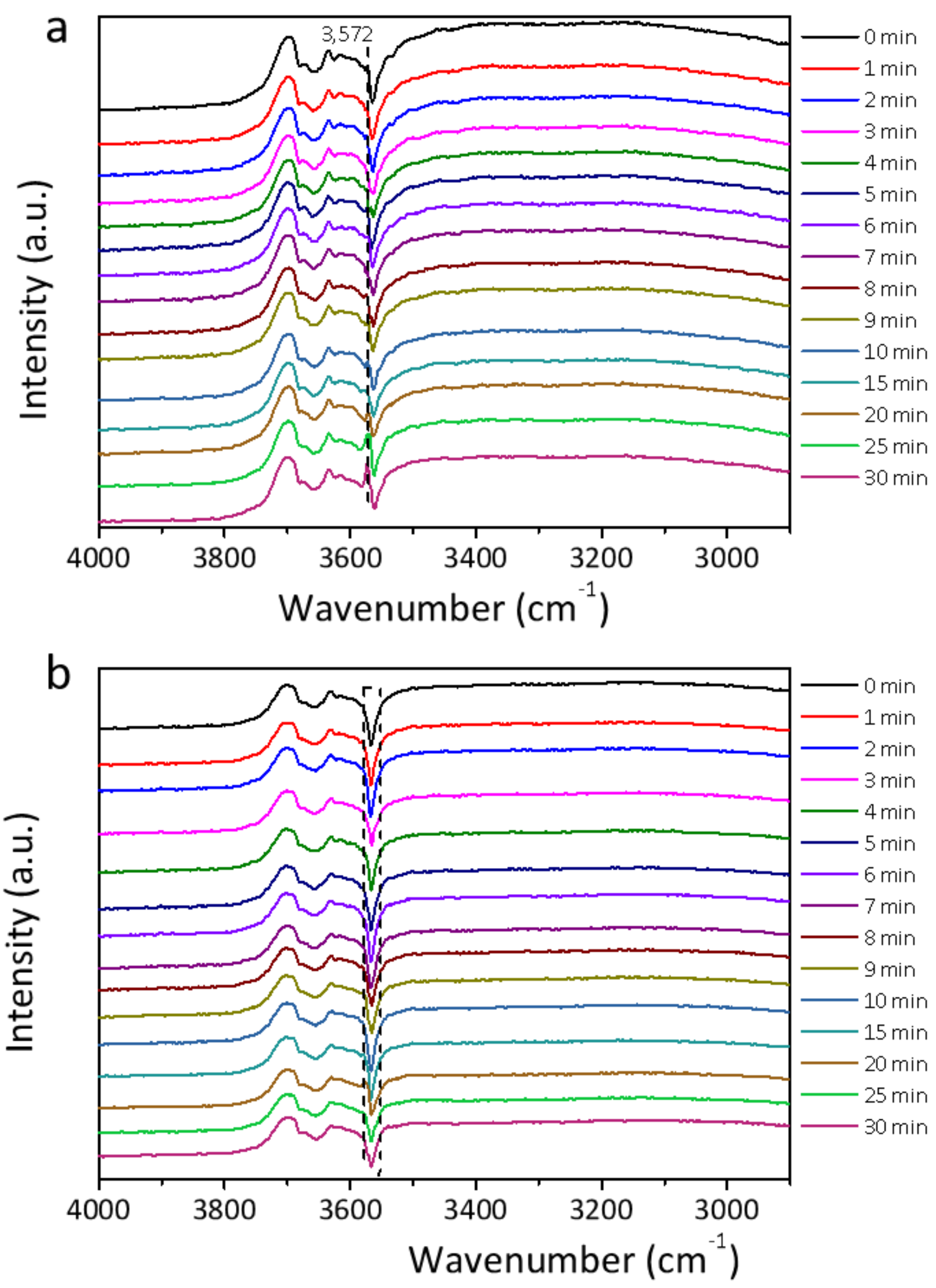

Figure S6. DRIFTS spectra obtained during exposure H_2 (1 sccm H_2, 19 sccm He) to 0.5 mol% Cu-HAP at a) 250 °C and b) 300 °C.

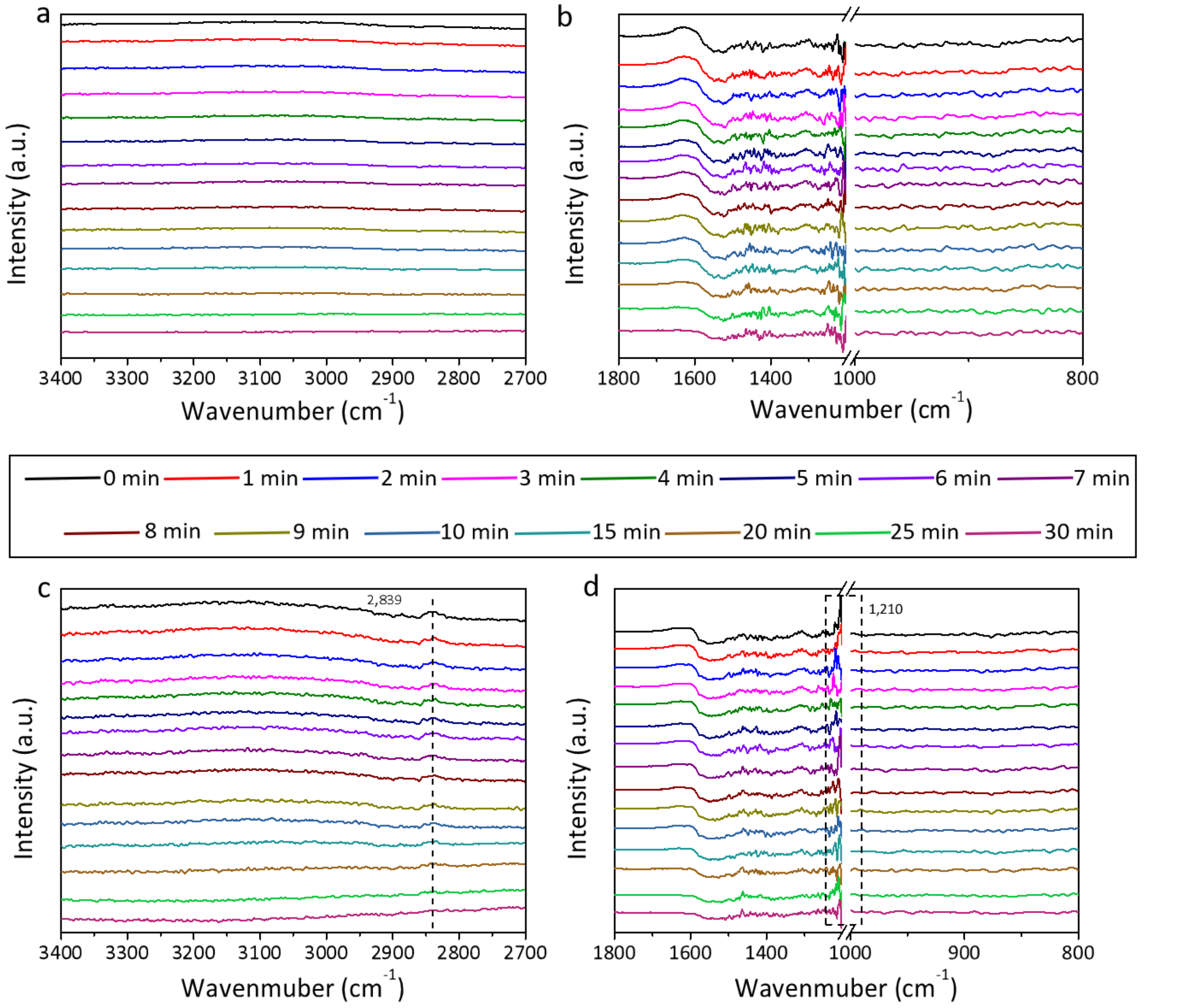

Figure S7. DRIFTS spectra obtained during 20 sccm He purging on a, b) 0 and c, d) 0.5 mol% Cu-HAP at 300 °C after H_2 absorption.

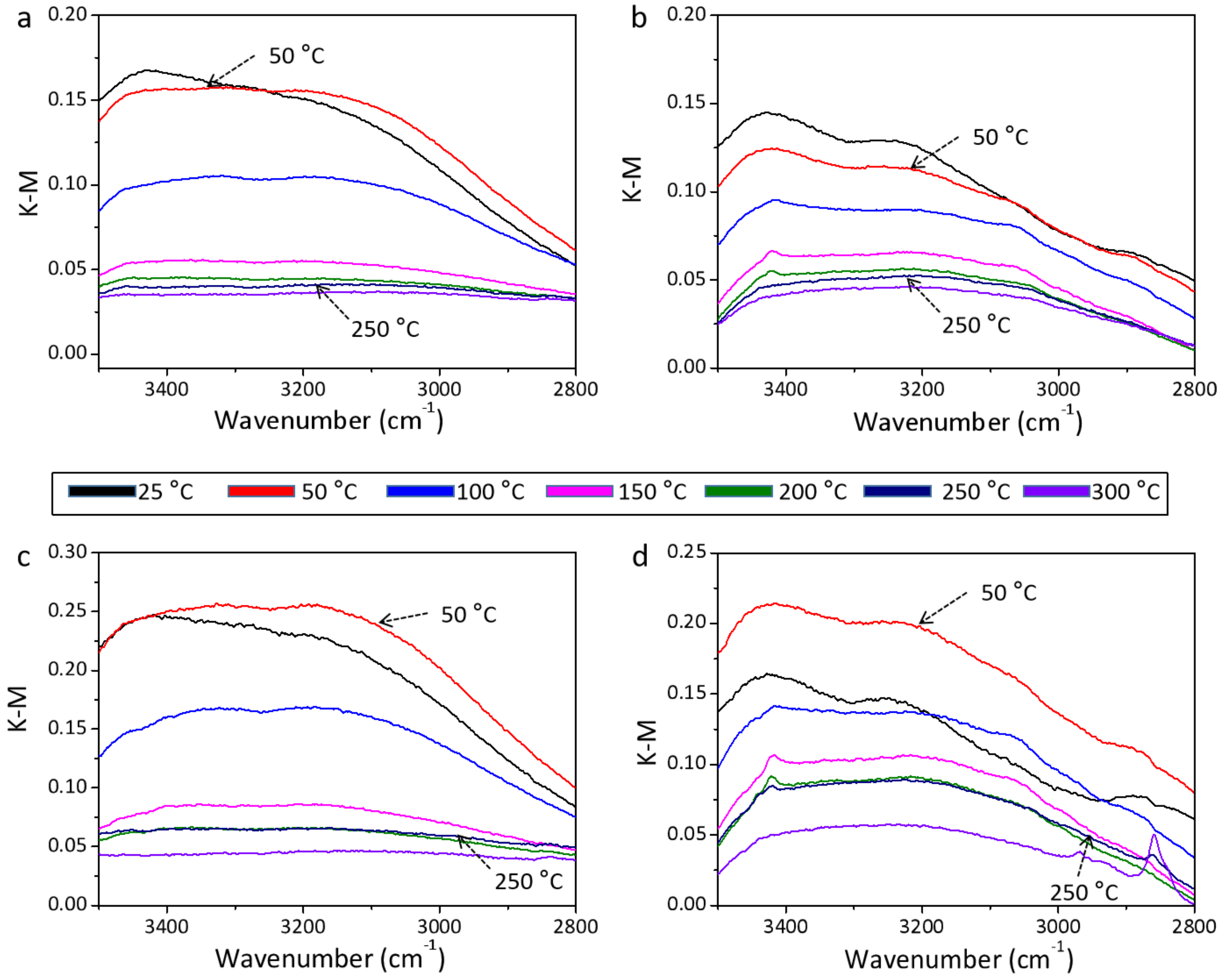

Figure S8. a, c) H_2 (1 sccm H_2, 19 sccm He) and b, d) H_2-CO_2 (1 sccm H_2, 1 sccm CO_2, 18 sccm He) adsorption spectra obtained over the range of 3500-2800 cm^{-1} on a, b) 0 and c, d) 0.5 mol% Cu-HAP after 30 min at each temperature.

Notably, on exposure 0.5 mol% Cu-HAP to either H_2 or H_2/CO_2, The peak growth in hydroxyl stretching and deformation region represented the enhanced H_2 adsorption and CO_2 activation with Cu substitution. Additionally, peak intensity gradually decreased with increasing temperature from 25 to 300 °C on 0 mol% Cu-HAP, while 0.5 mol% Cu-HAP displayed an increase of peak intensity at the temperatures of 50 and 250 °C, this is consistent well with two-stage linear plots in Arrhenius plots of 0.5 mol% Cu-HAP (Figure 1c).

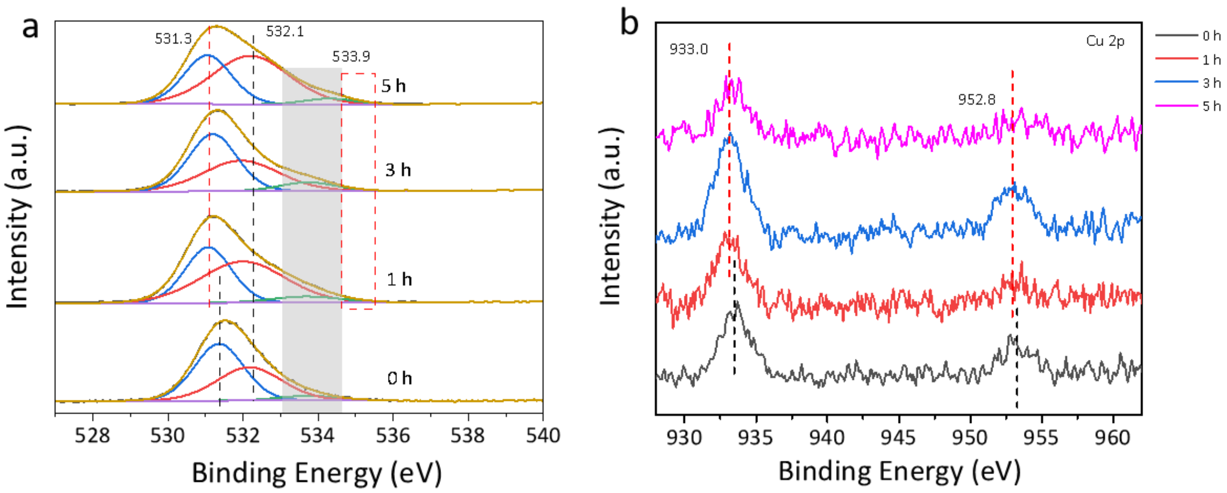

Figure S9. a) O 1s and b) Cu 2p XPS spectra were obtained by following exposure sample to a 1:1 ratio of CO_2/H_2 at 300 °C under light for 0, 1, 3 and 5h, respectively.

The O 1s XPS spectra could be fitted with three peaks around 531.3, 532.1 and 533.9 eV, which corresponded to lattice oxygen, oxygen vacancy and hydroxyl, respectively. The spin-orbit doublet Cu 2p core level XPS peaks around 933.0 eV and 952.8 eV attributed to Cu^{2+} sites in the HAP lattice.

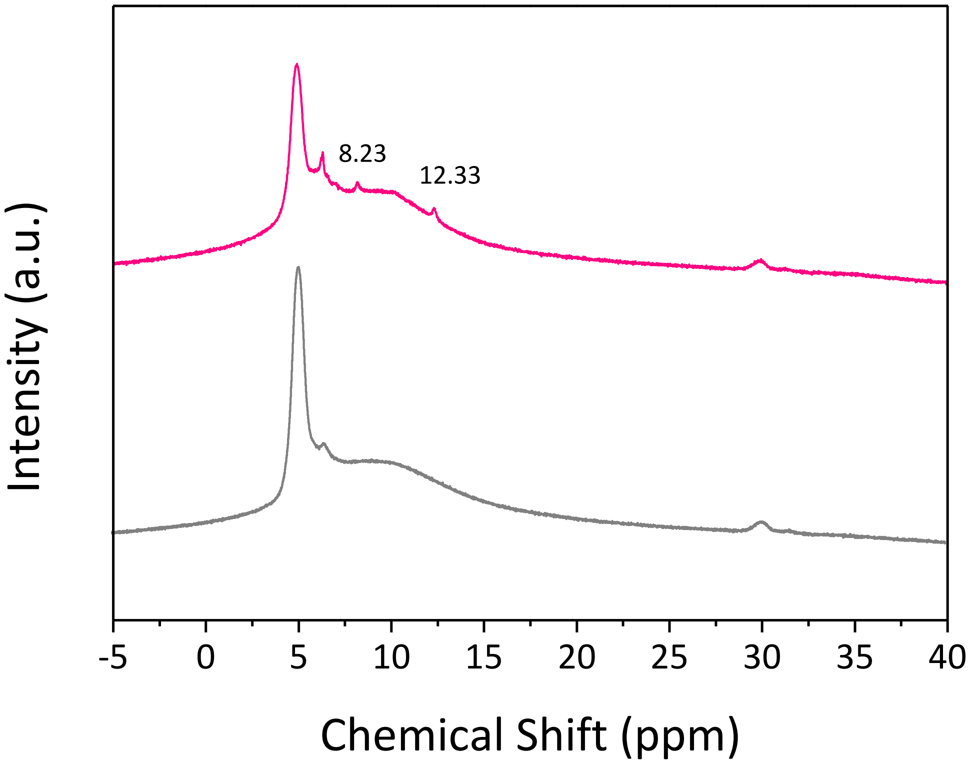

Figure S10. 1H MAS-NMR spectra of 0.5 mol% Cu-HAP before (gray) and after treated using H_2 at atmospheric pressure and 300 °C (pink).

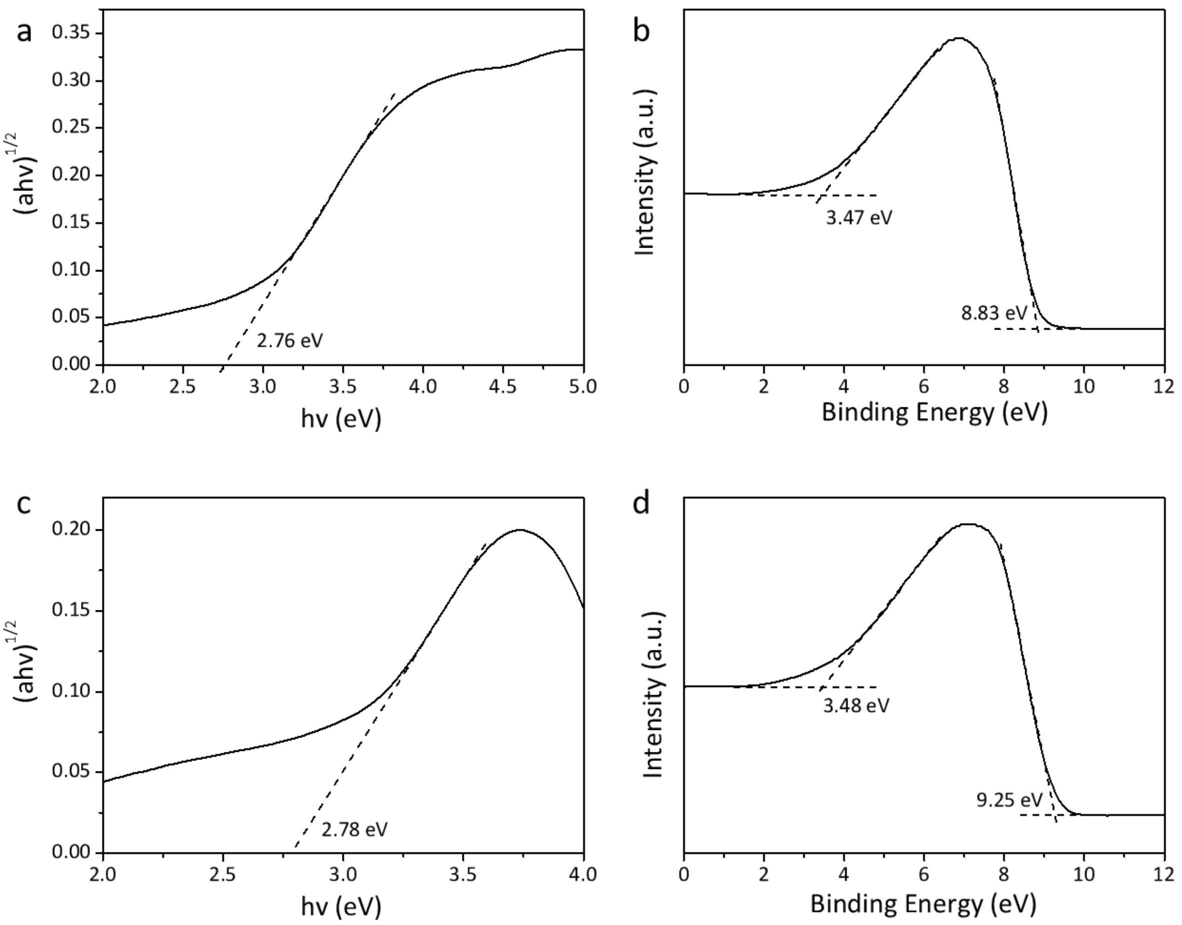

Figure S11. The band gap energy and UPS spectra of a, b) 0 and c, d) 0.5 mol% Cu-HAP.

The results established valence band and conduction band positions of -15.84 and -13.08 eV for 0 mol% Cu-HAP; and -15.33 and -12.55 eV for 0.5 mol% Cu-HAP.

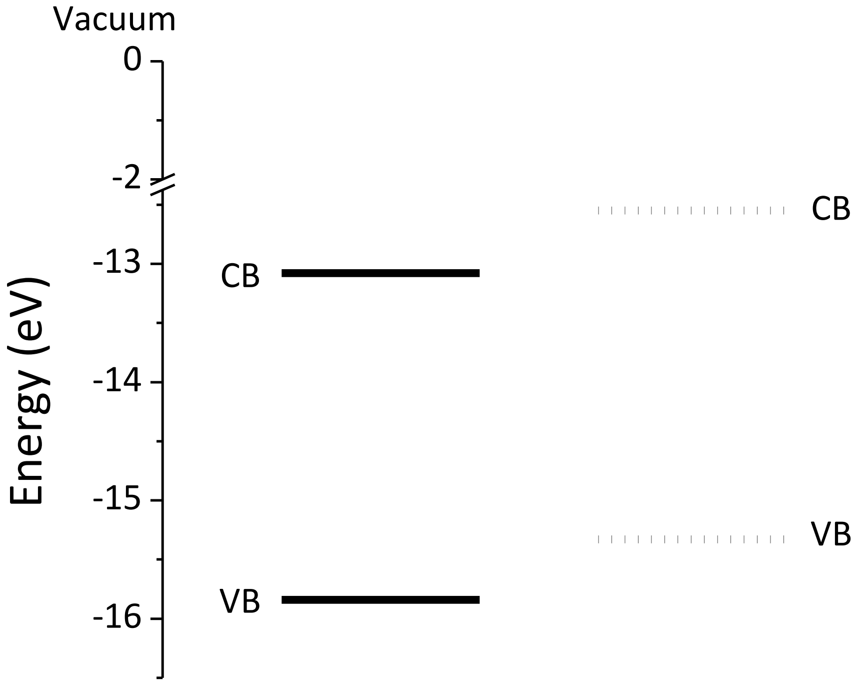

Figure S12. Illustration of the electronic band structure with the band energy diagrams of 0 (solid lines) and 0.5 mol% Cu-HAP (dashed lines).

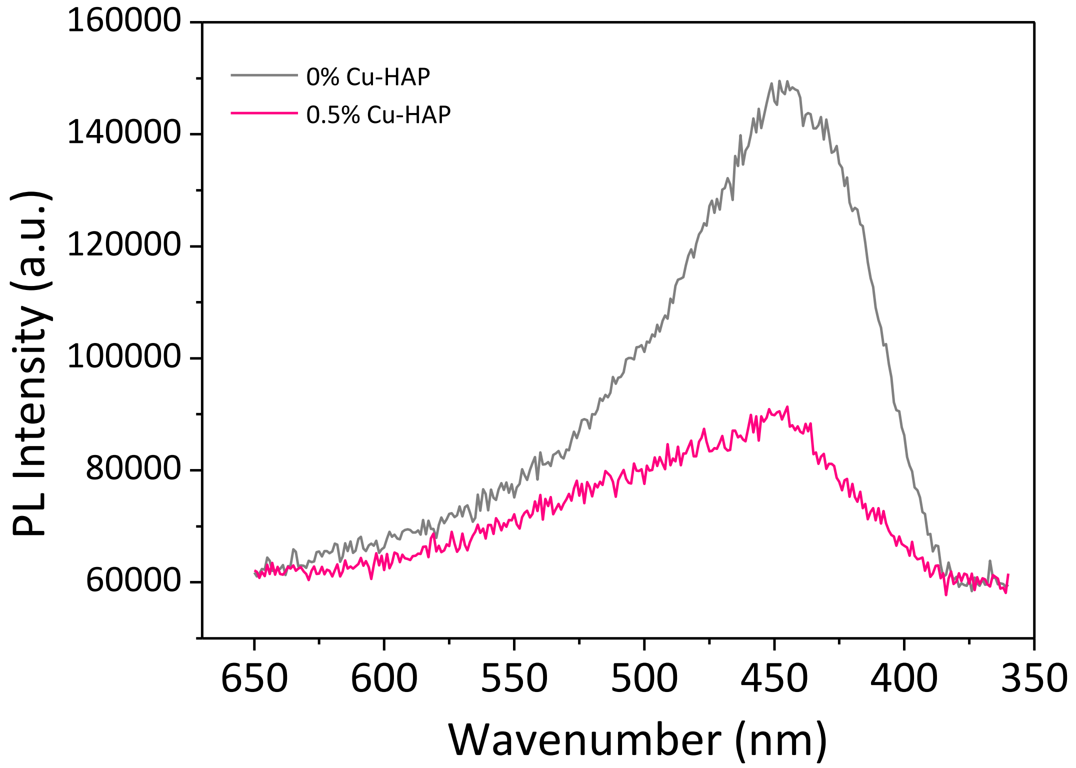

Figure S13. Photoluminescence (PL) spectra of 0 and 0.5 mol% Cu-HAP.

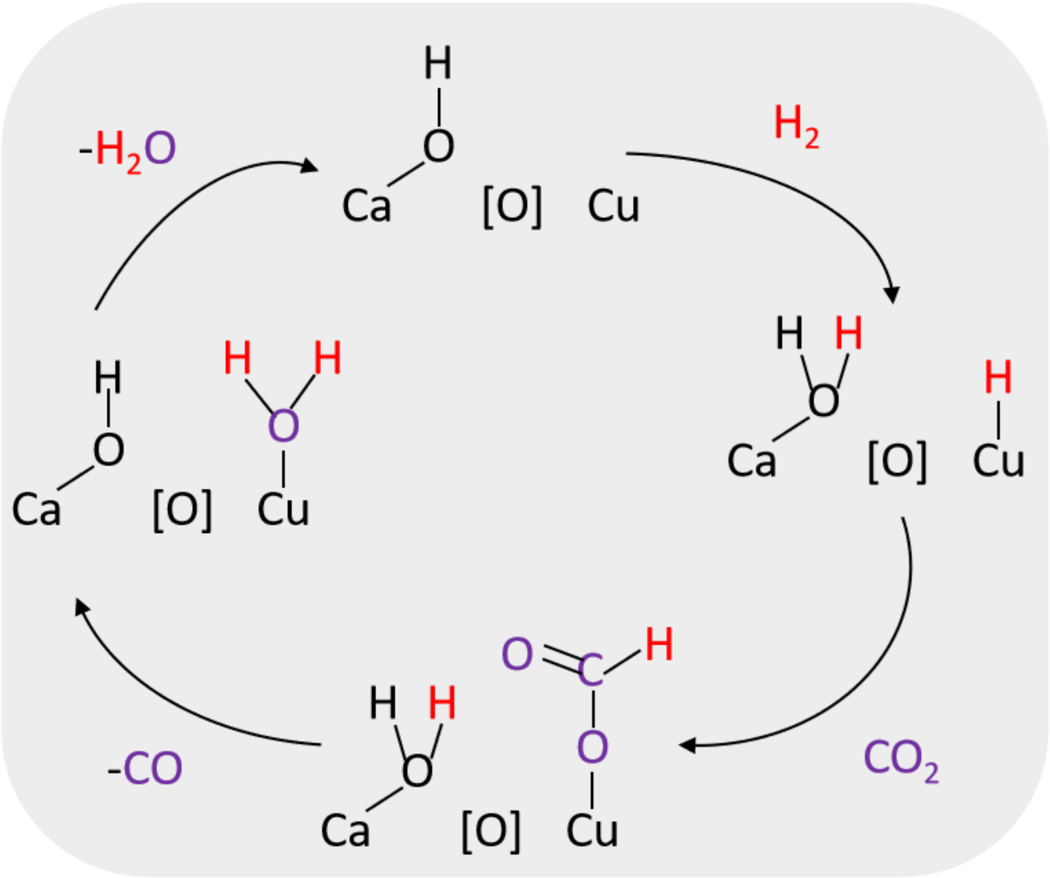

Figure S14. Reaction scheme for CO_2 hydrogenation to CO *via* the formate pathway on SFLPs in 0.5 mol% Cu-HAP.

Table S1. The average surface energy (γ) of (211), (112) and (300) surfaces. To examine the experimentally exposed active facet of HAP, surface energy of (211), (112) and (300) surfaces are calculated, according to the definition posed in previous literature.[2] Surface energies follow a sequence γ(112) > γ(300) > γ(211), indicating that the formation of (112) facet is more likely. We then conclude that the active reduction reaction is largely associated with (211) facet of HAP.

Facet	(211)	(112)	(300)
Surface Energy (J/m^2)	1.84	3.10	2.60

References

[1] J. Guo, P. N. Duchesne, L. Wang, R. Song, M. Xia, U. Ulmer, W. Sun, Y. Dong, J. Y. Y. Loh, N. P. Kherani, J. Du, B. Zhu, W. Huang, S. Zhang, G. A. Ozin, *ACS Catal.* **2020**, *10*, 13668.

[2] K. H. L. Zhang, A. Walsh, C. R. A. Catlow, V. K. Lazarov, R. G. Egdell, *Nano Lett.* **2010**, *10*, 3740.
